# CDKAM: a taxonomic classification tool using discriminative k-mers and approximate matching strategies

**DOI:** 10.1186/s12859-020-03777-y

**Published:** 2020-10-20

**Authors:** Van-Kien Bui, Chaochun Wei

**Affiliations:** 1 Department of Bioinformatics and Biostatistics, School of Life Sciences and Biotechnology, Shanghai Jiao Tong University, Shanghai, 200240 China; 2 Shanghai Center for Systems Biomedicine, Shanghai Jiao Tong University, Shanghai, 200240 China

**Keywords:** Third generation sequencing, Taxonomic classification, Discriminative k-mer, Approximate matching

## Abstract

**Background:**

Current taxonomic classification tools use exact string matching algorithms that are effective to tackle the data from the next generation sequencing technology. However, the unique error patterns in the third generation sequencing (TGS) technologies could reduce the accuracy of these programs.

**Results:**

We developed a Classification tool using Discriminative K-mers and Approximate Matching algorithm (CDKAM). This approximate matching method was used for searching k-mers, which included two phases, a quick mapping phase and a dynamic programming phase. Simulated datasets as well as real TGS datasets have been tested to compare the performance of CDKAM with existing methods. We showed that CDKAM performed better in many aspects, especially when classifying TGS data with average length 1000–1500 bases.

**Conclusions:**

CDKAM is an effective program with higher accuracy and lower memory requirement for TGS metagenome sequence classification. It produces a high species-level accuracy.

## Background

Metagenome sequencing is a powerful approach to study microbial communities in natural environments [1]. In a pipeline for the metagenomics project, taxonomic classification aims to accurately assign each fragment to its corresponding host organism and is one of the most important initial steps. With the progress of sequencing technology, modern metagenomics methods need to deal with vast sequence datasets. Identifying taxa for billions of reads according to a reference database with many thousands of microbial genomes available today is becoming a time-consuming process. As the database from NCBI is continuously growing and being more complete, we have to consider the trade-off between the size of the reference database and the classification accuracy as well as the computational cost.

Currently, there have been many methods developed to taxonomically classify metagenomic data. In general, they can be divided into two categories: (1) alignment-based methods and (2) sequence composition based methods, such as k-mer-based methods [2]. Alignment-based classifiers proceed by aligning metagenome sequences to all genomes in the reference database to find the genome with the best alignment. The most well-known alignment algorithm is BLAST program [3]. Although its original purpose was not for metagenomic classification, BLAST still works for this problem. Almost all classifiers extended from this method are accurate, however suffer from slow speed. Another type of alignment programs is fast for sequence mapping, such as Bowtie2 [4]. By applying a concatenation procedure to remove the shared regions between different strains of a species, Centrifuge [5] creates a highly compressed BWT-indexed reference database and achieves a higher speed. In contrast, sequence composition based methods, such as CLARK [6] and Kraken [7] are fast. The database of CLARK is basically a hash table, which contains a reduced set of k-mers and uses separate chaining to resolve collisions. Any k-mer that appears in more than one genome is removed. In Kraken, each k-mer is stored as a hash value and mapped to the lowest common ancestor of the source genome. Kraken 2 [8] improves upon Kraken by using a Compact Hash Table, a probabilistic data structure that supports storing only 15% of the database. The speed increases 5 times while maintaining the accuracy as the original version.

The advantages of the third-generation sequencing technologies such as long read length make them attractive for many applications including metagenomics study. With further improvement in throughput and error rate reduction, this platform can be a great promise for analyzing the structure of more complex microbial communities [9]. However, due to the noisy outputs (error rate of 10–20%) of TGS platforms such as Nanopore [10] or PacBio [11], current taxonomic classification methods for NGS sequencing data do not work well for TGS sequencing data in some situations. For example, under a simple binomial model on a read length of 1000 and a uniform 15% error rate, only 73 exact 16-mers matches are found in the comparison with its correct mapping location [12]. Metamaps [12] is a hybrid tool that combines mapping strategy based on short k-mer and a probabilistic model using Expectation Maximization (EM) algorithm to estimate the sample composition. Simulating on long reads, Metamaps provides a higher accuracy but a hundred times slower than other existing methods.

In this project, we present CDKAM, a new taxonomic classification tool for TGS sequencing data with high error rate. The efficiency of our method and other bioinformatics applications are compared and validated on simulated and real long-read metagenome sequencing data. The results show that CDKAM can classify TGS sequences to their source genomes accurately and efficiently.

## Implementation

### Design of k-mer data structure

**Fig. 1:**
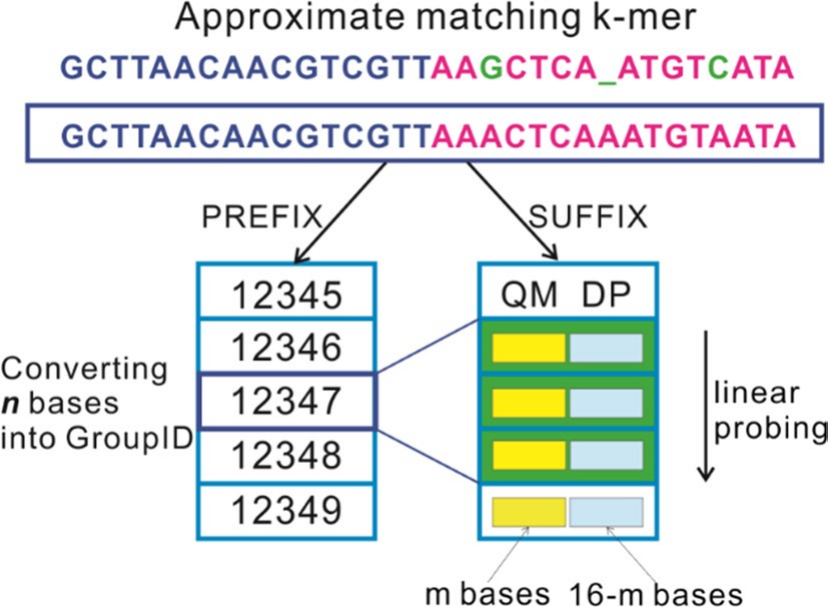
The structure of k-mers in CDKAM. A k-mer contains two parts: PREFIX and SUFFIX. SUFFIX can further be divided into two fragments: QM and DP, where QM stands for “quick mapping”, DP means “dynamic programming”, which are two fragments used for quick classification based on approximate matching strategies. For k-mers with the same PREFIX, we can group them by using a groupID converted from PREFIX, and save multiple k-mers (one PREFIX and multiple SUFFIXes) in a compressed way (see Reference database creation for more details)

We designed a data structure of k-mers as demonstrated in Fig. 1. A long k-mer with a size of 32 bases is chosen to increase the accuracy of classification. For each k-mer, the stored information includes a PREFIX (the first *n* bases, *n* could be equal or less than 16) and a SUFFIX (the last *16* bases). The SUFFIX is further divided into two parts: QM and DP with the length of *m* and *16-m*, correspondingly. Additionally, the SUFFIX is paired with the genus or species taxonomy ID of the k-mer. Here, we introduce an example about the approximate matching string used in CDKAM. GCTTAACAACGTCGTTAAGCTCA_ATGTCATA is approximately matched with GCTTAACAACGTCGTTAAACTCAAATGTAATA using the pink part, which contains two replacements and one deletion as the green highlighted characters in Fig. 1.

### Reference database creation

The reference genomes of archaea, bacteria, fungi, virus and human used for the clade exclusion experiments are downloaded from NCBI. As reported in May 2020, the total size is 84 GB.

**Fig. 2:**
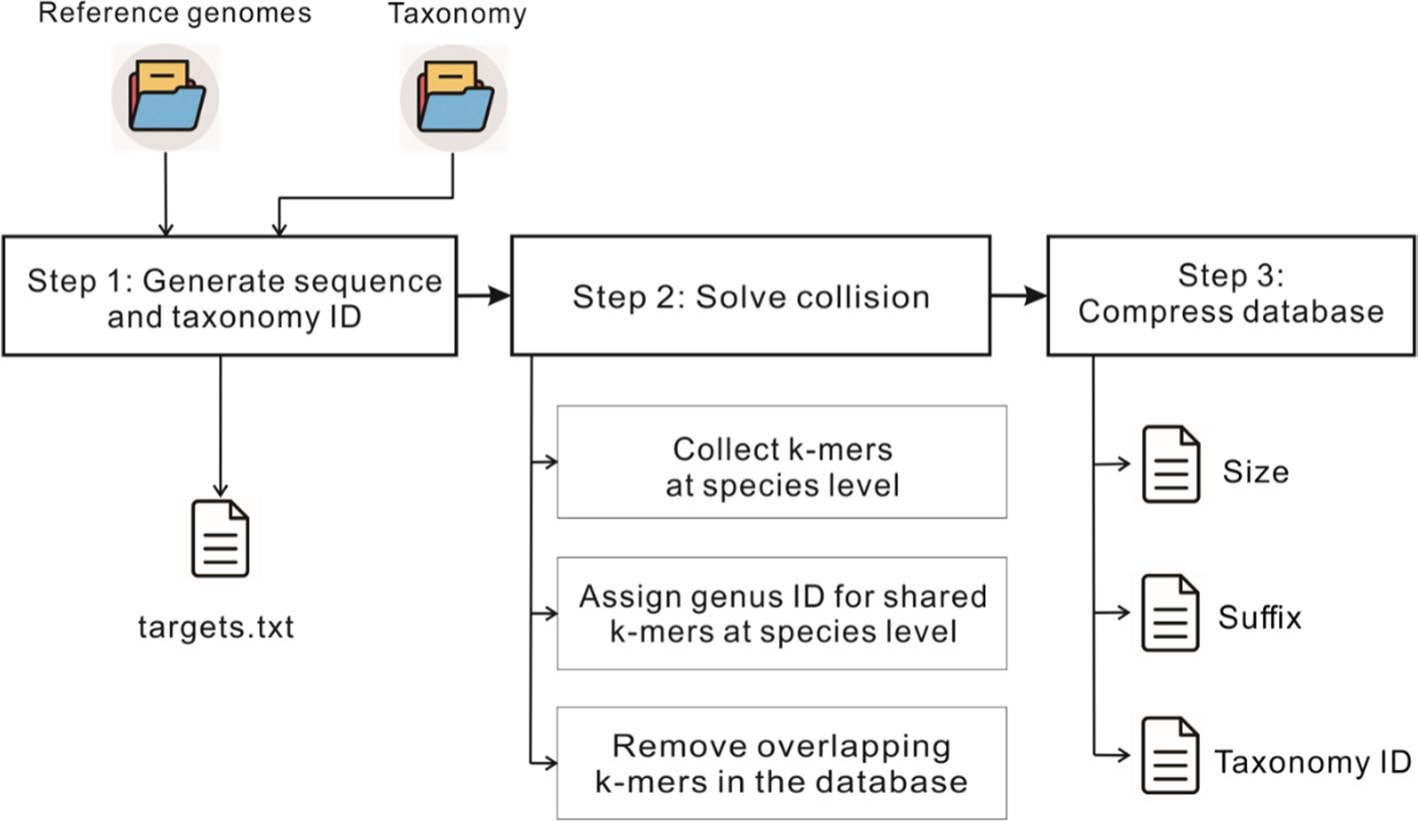
Diagram of reference database construction. Step 1: From the downloaded reference genomes and taxonomy information, CDKAM creates the mapping of sequences IDs and taxonomy IDs. Step 2: collecting k-mers and solving the k-mer collision to obtain discriminative k-mers as demonstrated in the Algorithm 1. Step 3: compressing the database. In the final step, the whole set of k-mers is divided into smaller groups. Then, for each group, CDKAM stores the number of kmers that shared the same group ID (saved in the Size file), the SUFFIX of k-mers (saved in the Suffix file) and their taxonomy ID (saved in the Taxonomy_ID file)

The database construction of CDKAM is shown in Algorithm 1 and Fig. 2. The k-mer collision is solved at three stages: species level, genus level and the whole database level. At the first step of collecting the k-mers of all strains that belong to a species, each identified k-mer is stored only once to avoid duplication. Next, the overlapping k-mers that are shared by at least two species of a genus are assigned to the taxonomy of the genus level. A proportion X% of the k-mers at each genus level is chosen (X% is set to 15% by default). They are selected uniformly over the range of each genome in order to maximize the chance of hits. After combining the k-mers from all the species used to construct the database, CDKAM removes any common k-mer between all genomes to obtain discriminative k-mers, which represent unique genomic regions characterizing each species. Finally, the full set is divided into 4^*n*^ smaller groups by using the integer value of k-mer’s PREFIX as the group ID. Consequently, CDKAM only need to store the size of each group, the SUFFIX and taxonomy ID of k-mers, which could reduce a large portion of memory consumption.

Algorithm 1: Description of CDKAM’s database building phase.
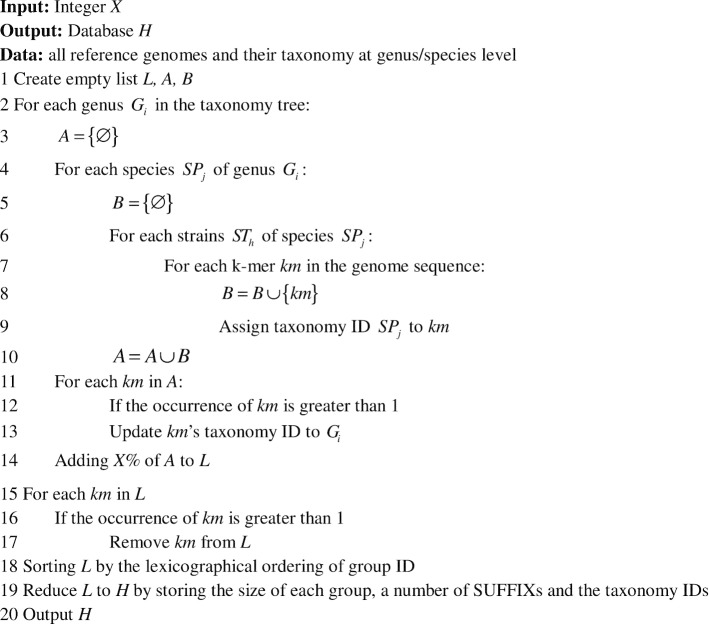


### Classification based on approximate mapping approach

Algorithm 2 presents the searching algorithm of CDKAM. For each k-mer of the querying read, the positions in the database that contain k-mers with the same group ID are examined. CDKAM uses a linear probe over this range, while Kraken and CLARK apply a binary search. During the searching procedure, an approximation algorithm is utilized to ignore at most 3 errors for a matched k-mer. The QM component with *m* bases in the SUFFIX of the querying k-mer and those in the database are mapped by a “Quick Matching” technique. Their converted integer values are masked by a seed mask such as 11,111*, 1111*1, … before comparing. The purpose of this step is to guarantee at least *m* *−* 1 bases are equal. With no more than one substitution, it can pass and move to the second check. The comparison of DP part is done by a banded “Dynamic Programming” algorithm with a width of 4. In this manner, we tolerate 2 errors, which can include insertions and deletions. The final assignment of a read to a taxonomy ID is determined by the highest number of matched k-mers.

Algorithm 2: CDKAM searching procedure.
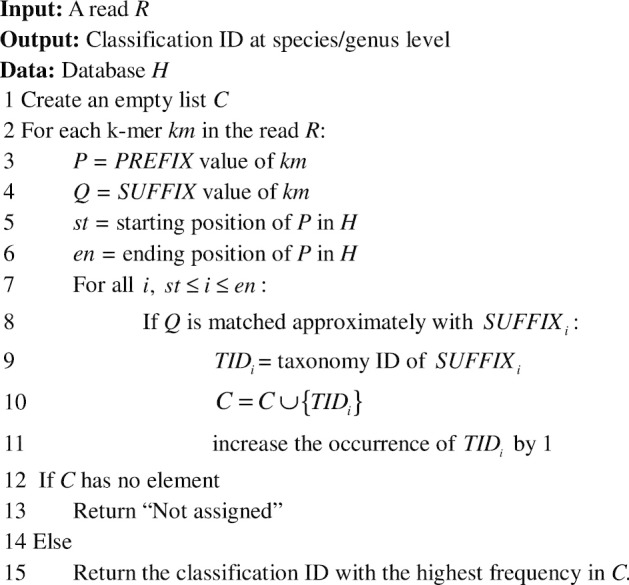


### Datasets for model selection

We compare Kraken 2, Centrifuge, CLARK, and CDKAM in different settings involving five simulated datasets and three real datasets.

Each simulated dataset contains multiple data. In the first scenario, we aim to assess the detection ability of classifiers when all sequencing reads were from genomes available in the reference database. For each bacterial genome in the reference database, 20 simulated reads are generated. The first dataset contains 342,360 reads from 17,118 bacterial genomes in the reference database. Next, we simulate a metagenome sample with 40 prokaryotic genomes as the simulation of Kraken2 [8] (Additional file 1: Table S5). For each genome, we generate 10,000 reads. There are totally 400,000 reads. The read length in both datasets is exactly 1000 bases. For each error rate at 5%, 10%, 15%, or 20%, sequencing errors are randomly added on the original reads (No. 1), and we call the resulted data No. 2, 3, 4, and 5 sample of the dataset respectively. Based on the same testing genomes, the third dataset contains five samples with 15% error rate, but the length of reads varies from 1000, 1500, 2000, 3000, and 4000 bases. The fourth dataset is created similarly to the third one with 20% error rate are added. The fifth simulation is carried out on a particular database and dataset. 70% of the bacterial genomes (4142 species) are chosen randomly and used for building the database, while the remaining part of the reference genomes is used for generating testing reads. For each strain among 1752 species in the testing set, 4000-bases long reads with 15% error rate are created.

Three sets of real metagenomic sequencing data are PRJNA493153 [13], Zymo R10 [14], and Zymo R10v2. The first one is a real Nanopore MinION sequencing data from the human microbe project PRJNA493153, which contains 548,721 reads with the average length of 1000 bases. There are 10 samples, the total size is 1.2 GB. Zymo R10 dataset is a Nanopore GridION sequencing data of the Zymo Community Standards R10 synthetic community, which comprises five Gram-positive bacteria, three Gram-negative bacteria, and two yeasts (Additional file 1: Table S6). There are more than 3 million reads with a total size of 25 GB. Additional file 1: Figure S1 presents statistics on the read lengths of Zymo dataset with the mean of 3860 bases. To generate a species-level true set, we use Minimap2 [15] with *-ax map-ont* to map the reads against the reference genomes provided by Zymo. All reads that cannot be mapped by Minimap2 are considered as the interference. For a read that can be mapped to multiple genomes by Minimap2, if a classifier provides the result matched with one of the possible taxonomy IDs, we will count as a true-positive classification. Zymo R10v2 dataset is modified from the Zymo R10 dataset in order to test classifiers against the TGS data sequences that have medium lengths. For each read, we trim 1500 bases in the middle region to create a new dataset.

### Evaluation of accuracy

To compare CDKAM with other tools, sensitivity, precision, and F1-scores are used and evaluated at genus or species level. The classified reads are grouped into the following categories: true-positive (TP), false-negative (FN), vague-positive (VP), and false-positive (FP). TP are those sequences assigned to the right genomes at the same taxonomy level or lower levels (species level is a lower level compared to genus level). A read belongs to the FN category if the classifier fails to assign the sequence. The concept of VP is used for the evaluation at species level only. A read classification is called a VP if it is assigned correctly only at genus level and does not have any further information about species level. Finally, an FP classification is defined as an incorrect classification, that is, not at the true genus nor true species of origin. The unclassified reads would belong to true-negative (TN) or false-negative (FN).

For example, a classification of an *Escherichia coli* fragment as *Escherichia* would be evaluated as a TP at genus level, but as a VP at species level. Classification of that same fragment as *Escherichia albertii* would be evaluated as FP at species level, but as a TP at genus level (because the LCA of *Escherichia albertii* and *Escherichia coli* is the genus taxon *Escherichia*).

The detection rate is the proportion of the positive classified reads vs the total number of reads, i.e. D-rate = (TP + FP + VP)/TOTAL. Sensitivity at a certain taxonomy level is defined as the proportion of the number of true-positive assignments vs the total number of reads classified, i.e. SEN = TP/(TP + FP + VP + FN). We define precision as the proportion of classifications that are true positives among the number of positive calls (excluding vague positives when evaluating at species level), i.e. PRE = TP/(TP + FP). An F1-score is computed as the harmonic mean of sensitivity and precision, i.e. F1 = 2 × SEN × PRE/(SEN + PRE).

### Computation environment

CDKAM and other taxonomic classifiers (Kraken2 v2.0.9, Centrifuge v1.0.4, CLARK v1.2.6.1) are benchmarked on a compute node having 512 GB of memory and Intel E5-2630 v4 processors. All experiments are run on single-thread mode. The database of all classifiers are updated to May 2020.

## Results and discussion

### Parameter settings

**Fig. 3:**
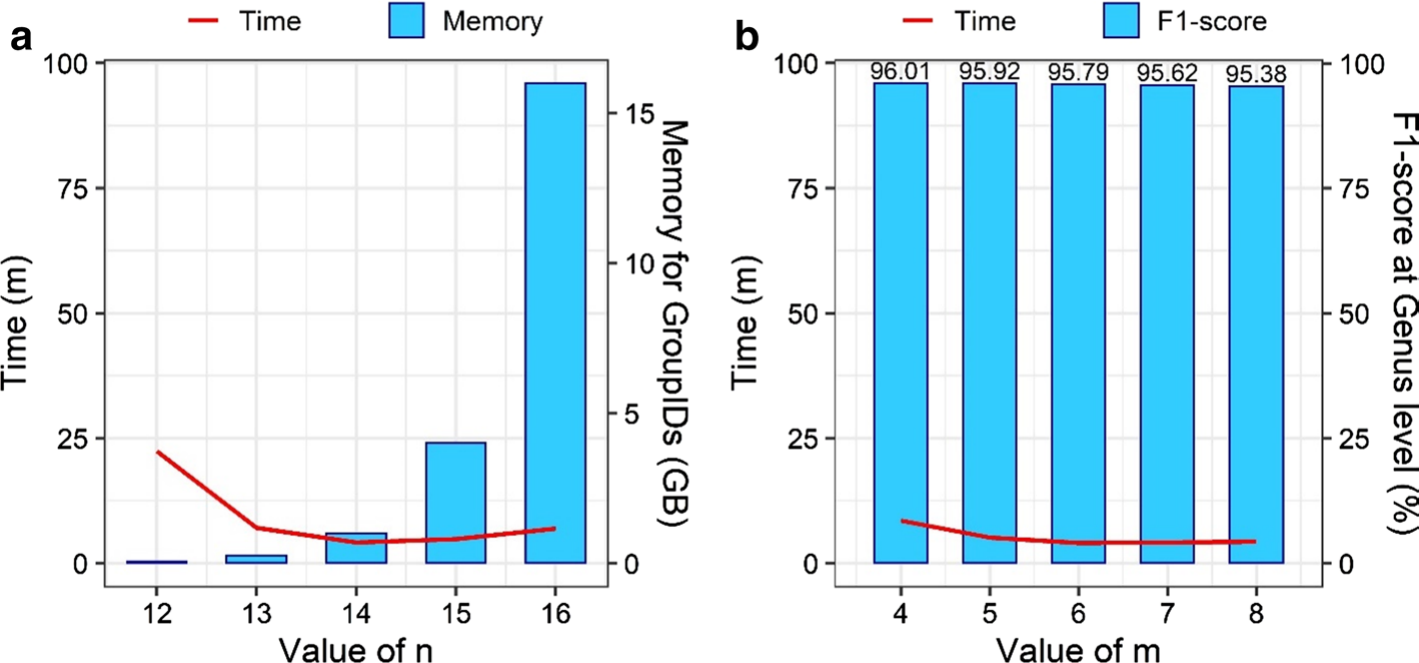
Performance of CDKAM with the different parameter settings. **a** *m* = 6 versus different values of *n*. **b** *n* = 14 with different values of *m*

By selecting the first *n* bases of k-mers as the PREFIX, the numbers showing the size of each group in the database require 4 × 4^*n*^ bytes. Besides that, the value of *m* during the searching query also influences the speed and efficiency of the CDKAM. In this part, we present the performance of X = 15% version with the different parameters of *n* and *m* on the sample No. 4 in the first dataset. Figure 3A presents the processing time and the extra memory needed for the group IDs when *m* = 6 and *n* varies. Meanwhile, Fig. 3B shows the processing time and the F1-scores at genus level with different values of *m* and a fixed *n* = 14. CDKAM tends to decrease the accuracy when *m* increases, but the difference is slight. Remarkably, the case of *m* = 6 is the optimal point of two comparison algorithms, quick mapping (QM) and dynamic programming (DP). From these experiments, we choose (*n* = 14,* m* = 6) to balance speed, memory usage, and accuracy.

The parameter X affects the size of the built database. A version with a greater value of X produces a larger database, which could decrease the speed but increase the accuracy. The difference in the accuracy of CDKAM with X = 10%, X = 15% and X = 20% is small, while that of CDKAM with X = 5% drops dramatically. More specific results are presented in Additional file 1: Table S1, Table S2, and Fig. 7. Unlike the collision avoidance algorithm in CLARK, which eliminates all intersections in the k-mer set of each species, the shared k-mers at the genus level in CDAKM are given a high priority and saved in the database. Consequently, it is unneeded to keep all k-mers in the final set. A fraction of the total k-mers can provide a good resolution about the true taxonomic origin of the sequencing data and also reduce the large computation cost.

### Performance on simulated data

**Fig. 4:**
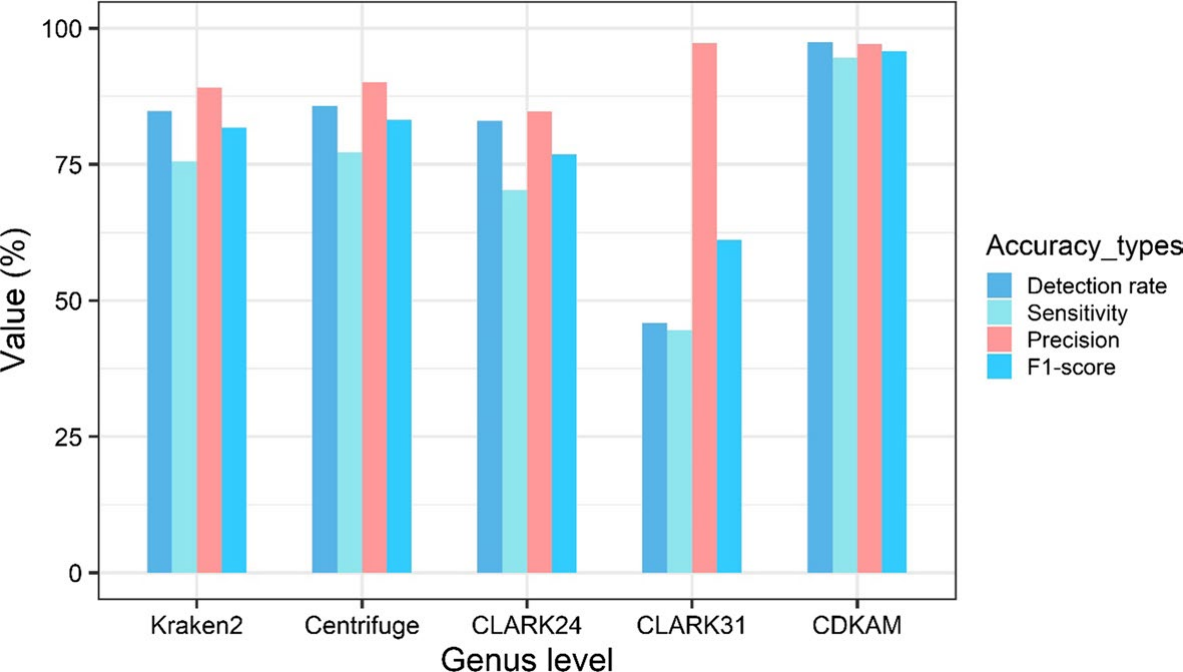
Comparison of methods on a data with a high sequencing error rate. Accuracy measurement types include detection rate, sensitivity, precision, and F1-score. The data used for the experiment is the sample No. 4 in the first dataset, which contains an error rate of 15%

When dealing with simulated reads without error, most tools give F1-scores greater than 90% at the genus level (Additional file 1: Table S1, Table S2). However, on the test cases containing sequencing errors, CDKAM achieves higher accuracy than any other classifier. For example, the default CDKAM version still exceeded an F1-score of 95.79% and 94.54% when classifying reads with 15% sequencing errors in the first and the second dataset. However, the accuracy of CLARK decreases significantly due to a low detection rate if a long k-mer (31-mers) is chosen. A shorter k-mer (24-mers) version gives a higher sensitivity, but the false positive would increase (Fig. 4). Similarly, Kraken 2 and Centrifuge also correctly classify a smaller amount of reads compared to CDKAM. This is caused by the exact string matching algorithm since many k-mers with one base variation could not be detected.

We use the third and the fourth experiment to assess the effect of read length on classification accuracy (Additional file 1: Table S3, Table S4). It is worth noting that the accuracy drops sharply between 15 and 20% error rate for all methods on the shorter reads. For example, on the read length 1000-bases, the F1-score of Kraken2 at genus level reduces significantly from 84.60 to 40.41%. By contrast, on the read length 4000-bases, the decrease is less serious. In this case, the gap is only 12.98%, from 97.89 to 84.91%. When the read length increases, there would be more hits that could provide more specific information about their true host organism. As a consequence, all methods perform better than they do on the shorter reads. Kraken2 and Centrifuge show a significant improvement with the F-1 score at genus level up to more than 97% when classifying the 4000-bases reads on the third dataset (Additional file 1: Table S3). It is noticeable that on the fourth dataset with a higher error rate at 20%, they perform worse than CDKAM (Additional file 1: Table S4). As opposed to a fall in the accuracy of other classifiers at species level, CDKAM with X = 20% still has a high F1-score in both simulations, which are 90.68% and 88.08%. These results indicate that CDKAM is more robust than other methods in the deal with the sequencing error, in particular for situations in which the error rate becomes higher.

The ability to deal with unknown sequences is an important concern in metagenomics, especially for those environmental samples with a high percentage of unknown species, which have no similar genome with a high sequence identity in the reference database. To address this, we include a fifth dataset by dividing the whole reference genomes of bacteria into two sets: 70% species for the reference database construction and the remaining 30% for testing, and then evaluating the accuracy at genus level. By selecting genomes randomly, the sequencing reads in the testing set could be originated from strains not represented in the reference database, or from those having siblings at family or genus level in the database. In this experiment, CDKAM detects 63.83% of the reads and then assign them to sibling species with those in the database. 37.7% of species have more than 50% classified reads, while 49.7% of species have more than 25% classified reads. In addition, 32.64% of species cannot be found, which means they have very different sequences and their k-mer sets do not intersect with those of other species. It is clear from these results that classifying unknown sequences is still a challenge for CDKAM.

### Performance on real data

**Fig. 5:**
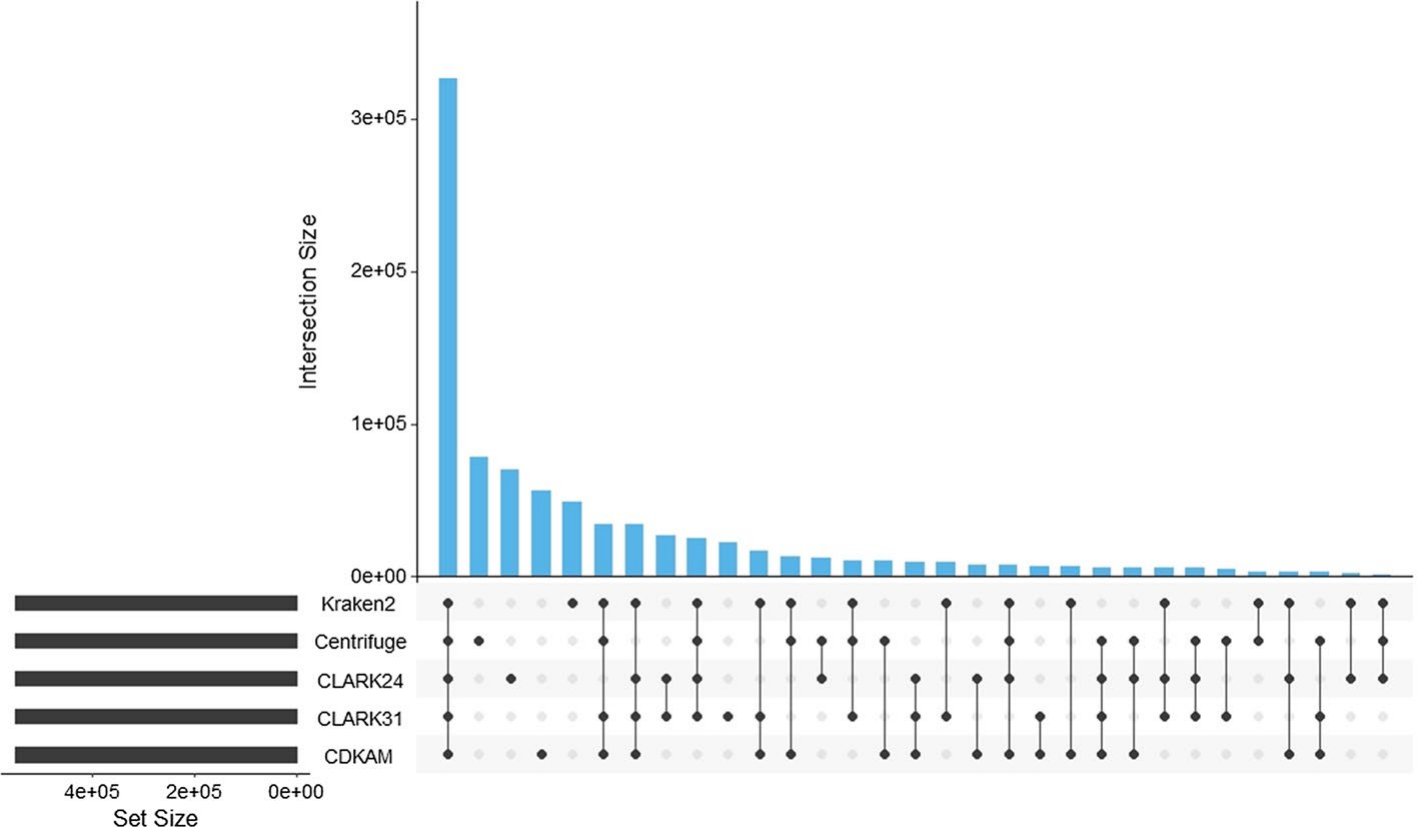
Comparison between CDKAM and other classification tools on the PRJNA493153 dataset. This figure is created using upset [16] (https://caleydo.org/tools/upset/) to show the intersection of results from different classification tools

CDKAM with X = 15% is selected to perform the evaluation on the real TGS data. On the PRJNA493153 dataset, CDKAM shows the highest consistency (Fig. 5 and Additional file 1: Figure S2). The detection rate of CDKAM, Kraken2, Centrifuge, CLARK24 and CLARK31 are 59.77%, 55.32%, 57.99%, 62.80% and 44.41%, correspondingly. Because there is no benchmark for this microbe community, we count how many reads getting the same genus-level taxonomy ID between different methods. There are 44.36% classified reads by CDKAM sharing the result with Kraken2 and 42.26% of them sharing the result with Centrifuge.

**Fig. 6:**
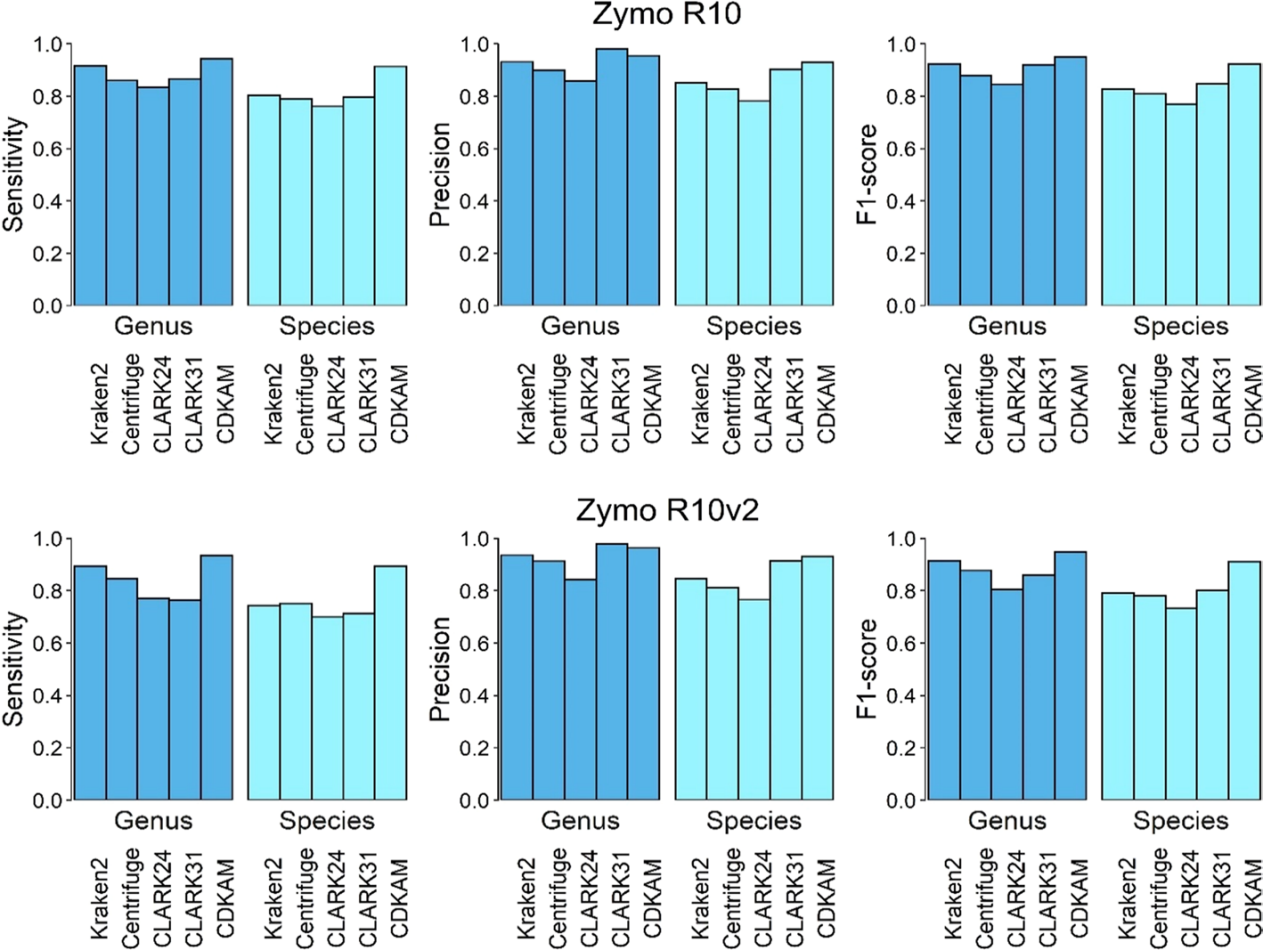
Comparison of the accuracy of classification methods on a real Nanopore (Zymo R10) dataset

The evaluation at the genus level on the Zymo R10 dataset indicates that the sensitivity of the CDKAM and Kraken2 assignments is higher than that of the remaining tools, while CLARK using 31-mers provides the highest precision (Fig. 6). The F1-score of CDKAM at genus level is 94.91% and slightly greater than that of CLARK and Kraken2, which are 92.31% and 92.02%, respectively. Besides, CDKAM performs the best at species level. The compositional estimation by CDKAM is the closet to alignment stats by Minimap2 as presented in Additional file 1: Table S7. Turning to the smaller dataset Zymo R10v2, almost all classifiers show a trend towards lower classification accuracy by 2–4% for shorter reads. Meanwhile, CDKAM performs stably and achieves the F1-score of 91.19% at the species level.

### Runtime and memory consumption

**Fig. 7:**
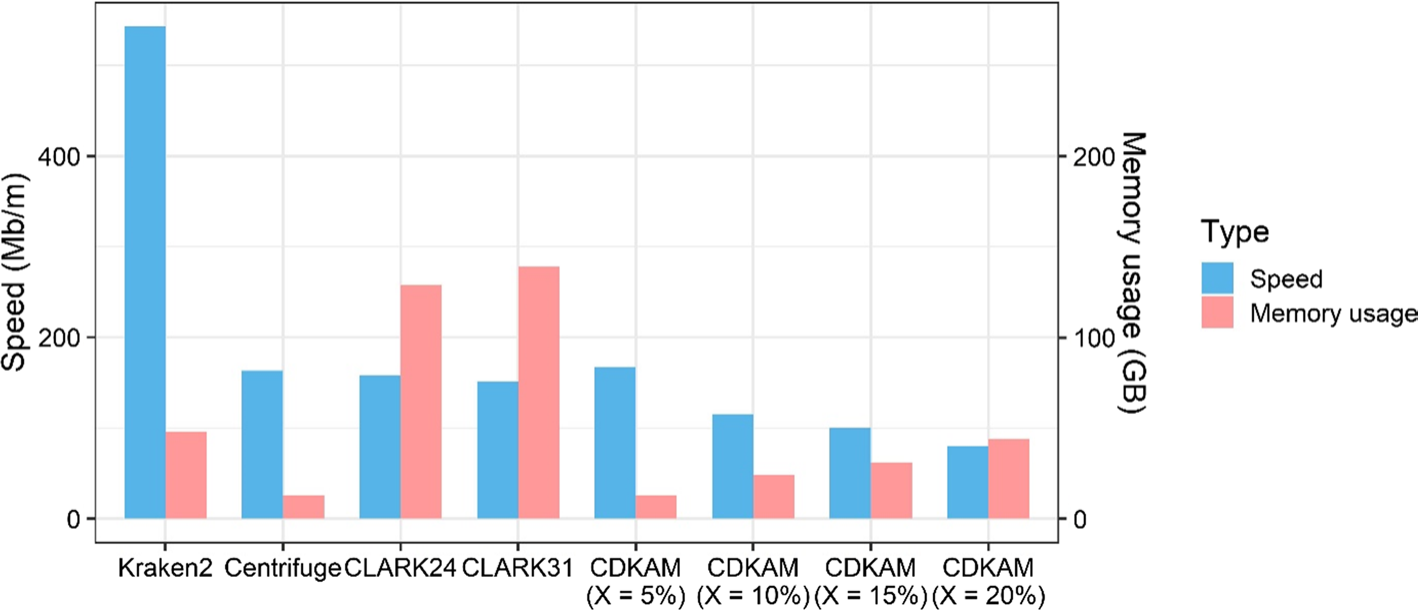
Comparision of speed (million bases per minutes) and memory consumption (GB) of classifiers on the Zymo R10 dataset

We use the Zymo R10 dataset to measure the actual processing speed of classifiers. As shown in Fig. 7, Kraken2 is ultrafast, whereas Centrifuge, CLARK and CDKAM could be grouped together in terms of speed. CDKAM is slower than Centrifuge, but the difference is negligible. Although the approximation algorithm is slower than the exact matching algorithm in general, a small and sufficient database reinforces CDKAM to speed up. In fact, CDKAM is slightly faster than CLARK in the case of classifying a medium size dataset such as PRJNA493153 data. For an experimental dataset of 100,000 TGS reads, CDKAM will finish the sequence classification in about 5 min.

CDKAM supports parallel processing by using thread pools mechanism. Each local thread reads a fragment of the input file, save it temporarily in the buffer and then wait in the queue. After finishing the reading process of N fragments, N threads carry out the classification parallelly. The classification results from these threads are finally merged into the output file. The wall-clock time of CDKAM (X = 15%) when running with 1, 2, 4, 8, and 16 threads on the Zymo R10 dataset are 129, 58, 32, 17, and 10 min, respectively.

### Novelty of CDKAM

The key novelty of CDKAM is the combination of an effective collision avoidance mechanism for building the database and approximate matching strategies for searching k-mers. It can deal with high sequencing error rates in TGS data and maintain a high speed as well. To achieve these purposes, a small portion of the final k-mer set is stored in the database of CDKAM, which can decrease the memory consumption and increase the processing speed, while approximate matching strategies with two phases of quick mapping and dynamic programming algorithm provide a higher opportunity to detect the alignment of query sequences and the database.

## Conclusions

CDKAM is a new taxonomy classification tool designed for sequencing reads with high error rates. Compared to existing tools, it has good speed, small memory requirement and high accuracy. Especially, it is more accurate than all other existing tools for reads with medium lengths, such as 1000–1500 bases. For very long sequencing reads, its accuracy performance is comparable to the best existing tools, such as Kraken2 and Centrifuge.

## Availability and requirements

Project name: CDKAM

Project home page: https://github.com/SJTU-CGM/CDKAM

Operating system(s): Linux

Programming language: Perl, C++ 11, and Shell

Other requirements: Perl (5.10.1 or above), G++ (4.8.5 or above)

License: GNU GPL v.3

Any restrictions to use by non-academics: None

## Supplementary information

**Additional file 1.** Supplementary file for Table S1-7 and Figure S1-2.

## Data Availability

All simulated data in this paper is available in https://github.com/SJTU-CGM/CDKAM. The Zymo data are publicly available (https://github.com/LomanLab/mockcommunity)

## Abbreviations

BWT: Burrows–Wheeler transform
NCBI: National Center for Biotechnology Information
NGS: The next generation sequencing
TGS: The third generation sequencing
