# Supplementary material for: CDKAM: a taxonomic classification tool using discriminative k-mers and approximate matching strategies
Source: BMC Bioinformatics. 2020 Oct 20;21:468. doi: 10.1186/s12859-020-03777-y (PMC7576720; doi:10.1186/s12859-020-03777-y)
Supplement: Supplementary file 1 — Additional file 1. Supplementary file for Table S1-7 and Figure S1-2. [file 12859_2020_3777_MOESM1_ESM.docx]

**Additional file 1**

**CDKAM: a taxonomic classification tool using discriminative k-mers and approximate matching strategies**

Authors: Van-Kien Bui^1^, Chaochun Wei^1,2*^

^1^Department of Bioinformatics and Biostatistics, School of Life Sciences and Biotechnology, Shanghai Jiao Tong University, Shanghai 200240, China

^2^Shanghai Center for Systems Biomedicine, Shanghai Jiao Tong University, Shanghai 200240, China

*Email: [ccwei@sjtu.edu.cn](mailto:ccwei@sjtu.edu.cn)

Associate Editor: XXXXXXX

Received on XXXXX; revised on XXXXX; accepted on XXXXX

This file contains 7 Tables and 2 Figures.

Table S1. Performance comparison of Kraken 2, Centrifuge, CLARK and CDKAM on the first dataset.

Table S2: Performance comparison of Kraken 2, Centrifuge, CLARK and CDKAM on the second dataset.

Table S3: Performance comparison of Kraken 2, Centrifuge, CLARK and CDKAM on the third dataset.

Table S4: Performance comparison of Kraken 2, Centrifuge, CLARK and CDKAM on the fourth dataset.

Table S5: Reference genomes used for the second, the third and the fourth dataset. This list is referred from the experiment in Kraken 2.

Table S6: The list of 10 species were presented Zymo mock community R10.

Table S7: The number of reads classified by different classifiers in the Zymo mock community R10.

Figure S1: The distribution of read length in Zymo R10 dataset.

Figure S2: Comparison of classifiers on the PRJNA493153 dataset by measuring the percentage of reads that have the same classification results.

| **Software** | **No. 1**  **Original reads** | **No. 2, 5%**  **error rate** | **No. 3, 10%**  **error rate** | **No. 4, 15%**  **error rate** | **No. 5, 20%**  **error rate** |
| --- | --- | --- | --- | --- | --- |
| Kraken 2 | **97.25**  91.24 | **97.70**  88.88 | 95.78  82.85 | 81.79  64.79 | 38.98  27.48 |
| Centrifuge | 96.97  90.27 | 97.24  89.91 | 95.37  84.08 | 83.13  67.75 | 51.41  39.02 |
| CLARK 21-mers | 93.97  93.97 | 87.63  85.12 | 80.45  77.71 | 68.10  65.66 | 43.36  41.36 |
| CLARK 24-mers | 94.12  94.12 | 91.64  88.44 | 86.41  82.58 | 76.80  73.23 | 53.71  50.81 |
| CLARK 27-mers | 94.21  94.21 | 92.85  89.84 | 87.78  84.37 | 74.85  71.97 | 43.93  42.04 |
| CLARK 31-mers | 94.31  **94.31** | 92.70  **90.18** | 85.72  83.10 | 61.14  59.26 | 24.30  23.44 |
| CDKAM (X = 5%) | 97.32  92.27 | 96.93  85.30 | 96.68  83.64 | 86.43  67.69 | 51.70  31.70 |
| CDKAM (X = 10%) | 97.07  92.49 | 97.03  85.62 | 97.16  85.40 | 94.08  78.80 | 69.30  49.68 |
| CDKAM (X = 15%) | 97.17  92.40 | 97.32  85.76 | **97.46**  **85.85** | 95.79  81.44 | 76.16  57.57 |
| CDKAM (X = 20%) | 96.69  91.94 | 96.84  85.26 | 97.02  85.62 | **96.07**  **82.67** | **81.29**  **64.20** |

**Table S1: Performance comparison of Kraken 2, Centrifuge, CLARK and CDKAM on the first dataset.** The dataset contains 342,360 reads from 17,118 bacterial genomes in the reference database. 20 simulated reads with the length of 1000 bases are generated for each genome. F1-scores of classifiers at genus and species levels were shown. In each cell, the first number in a cell represents F1-score at genus level, and the second number is F1-score at species level. The numbers in bold are for the best performance for a data (in a column). Results here show that CDKAM performs much better than other tools when the sequencing error rate is closer to the real sequencing error rate.

| **Software** | **No. 1**  **Original reads** | **No. 2, 5%**  **error rate** | **No. 3, 10%**  **error rate** | **No. 4, 15%**  **error rate** | **No. 5, 20%**  **error rate** |
| --- | --- | --- | --- | --- | --- |
| Kraken 2 | 99.32  90.94 | 98.53  89.80 | 97.55  85.71 | 84.56  68.03 | 40.30  28.92 |
| Centrifuge | **99.68**  92.43 | **98.78**  **91.10** | **97.80**  86.97 | 87.02  71.35 | 54.76  41.76 |
| CLARK 21-mers | 93.19  93.12 | 88.43  85.98 | 83.76  81.38 | 71.13  68.95 | 43.48  41.69 |
| CLARK 24-mers | 93.27  93.19 | 92.30  89.10 | 89.07  85.69 | 80.07  76.71 | 55.76  52.96 |
| CLARK 27-mers | 93.31  93.24 | 93.12  90.25 | 89.92  86.96 | 77.51  74.73 | 45.62  43.71 |
| CLARK 31-mers | 93.35  **93.28** | 92.91  90.55 | 88.11  85.81 | 63.46  61.64 | 25.31  24.41 |
| CDKAM (X = 5%) | 96.74  91.81 | 96.06  88.43 | 95.68  86.78 | 85.24  69.95 | 47.99  30.94 |
| CDKAM (X = 10%) | 96.78  92.17 | 96.18  88.40 | 96.16  88.36 | 92.98  81.15 | 67.09  50.06 |
| CDKAM (X = 15%) | 96.88  92.31 | 96.32  88.33 | 96.31  88.67 | 94.54  83.69 | 74.56  58.46 |
| CDKAM (X = 20%) | 96.98  92.31 | 96.34  88.21 | 96.43  **88.82** | **95.26**  **85.34** | **80.11**  **65.50** |

**Table S2: Performance comparison of Kraken 2, Centrifuge, CLARK and CDKAM on the second dataset.** The dataset contains 400,000 reads from 40 bacterial genomes as listed in Table S5. 10,000 simulated reads with the length of 1000 bases are generated for each genome. The accuracy measurements are the same as in Table S1.

| **Software** | **No. 1, Length**  **= 1000** | **No. 2, Length = 1500** | **No. 3, Length**  **= 2000** | **No. 4, Length = 3000** | **No. 5, Length = 4000** |
| --- | --- | --- | --- | --- | --- |
| Kraken 2 | 84.60  68.20 | 92.45  77.25 | 95.55  81.68 | **97.42**  85.75 | **97.89**  87.61 |
| Centrifuge | 87.14  71.54 | 92.82  78.36 | 95.36  82.15 | 97.16  85.94 | 97.78  87.78 |
| CLARK 21-mers | 71.10  68.93 | 76.53  74.43 | 79.58  77.55 | 82.77  80.92 | 84.44  82.74 |
| CLARK 24-mers | 80.13  76.76 | 84.28  80.65 | 86.52  82.72 | 89.01  85.07 | 90.47  86.42 |
| CLARK 27-mers | 77.56  74.79 | 83.91  80.73 | 87.05  83.61 | 90.10  86.36 | 91.61  87.67 |
| CLARK 31-mers | 63.63  61.80 | 73.77  71.55 | 79.64  77.15 | 85.82  82.96 | 88.71  85.63 |
| CDKAM (X = 5%) | 85.34  70.10 | 91.88  79.56 | 94.30  83.90 | 95.86  87.59 | 96.27  89.08 |
| CDKAM (X = 10%) | 93.03  81.11 | 95.39  85.90 | 96.04  87.84 | 96.46  89.61 | 96.59  90.32 |
| CDKAM (X = 15%) | 94.56  83.78 | 95.96  87.21 | 96.35  88.69 | 96.62  89.98 | 96.71  90.59 |
| CDKAM (X = 20%) | **95.28**  **85.39** | **96.19**  **87.98** | **96.51**  **89.17** | 96.72  **90.22** | 96.81  **90.68** |

**Table S3: Performance comparison of Kraken 2, Centrifuge, CLARK and CDKAM on the third dataset.** The dataset contains 400,000 reads from 40 bacterial genomes as listed in Table S5. 10,000 simulated reads with 15% error rate are generated for each genome. The accuracy measurements are the same as in Table S1.

| **Software** | **No. 1, Length**  **= 1000** | **No. 2, Length = 1500** | **No. 3, Length**  **= 2000** | **No. 4, Length = 3000** | **No. 5, Length = 4000** |
| --- | --- | --- | --- | --- | --- |
| Kraken 2 | 40.41  28.97 | 54.35  40.30 | 64.43  49.14 | 77.44  61.38 | 84.91  69.13 |
| Centrifuge | 54.81  41.76 | 65.90  51.20 | 73.15  57.66 | 82.17  66.44 | 87.32  72.03 |
| CLARK 21-mers | 43.50  41.75 | 51.18  49.48 | 56.07  54.43 | 61.97  60.45 | 65.26  63.89 |
| CLARK 24-mers | 55.81  52.99 | 64.41  61.17 | 69.83  66.34 | 76.39  72.64 | 80.17  76.34 |
| CLARK 27-mers | 45.52  43.73 | 56.86  54.50 | 64.56  61.75 | 74.10  70.77 | 79.54  75.88 |
| CLARK 31-mers | 25.43  24.54 | 34.58  33.36 | 42.07  40.57 | 53.44  51.50 | 61.38  59.09 |
| CDKAM (X = 5%) | 48.04  30.99 | 62.96  44.78 | 72.66  55.02 | 83.57  68.15 | 88.93  75.53 |
| CDKAM (X = 10%) | 67.17  50.08 | 79.77  63.82 | 86.37  72.03 | 92.35  80.75 | 94.58  84.77 |
| CDKAM (X = 15%) | 74.59  58.51 | 85.30  70.78 | 90.35  77.48 | 94.36  84.04 | 95.71  86.94 |
| CDKAM (X = 20%) | **80.33**  **65.77** | **88.81**  **75.90** | **92.45**  **81.10** | **95.14**  **85.94** | **96.02**  **88.08** |

**Table S4: Performance comparison of Kraken 2, Centrifuge, CLARK and CDKAM on the fourth dataset.** The dataset contains 400,000 reads from 40 bacterial genomes as listed in Table S5. 10,000 simulated reads with 20% error rate are generated for each genome. The accuracy measurements are the same as in Table S1.

| **No** | **NCBI TaxID** |  | **Taxonomic lineage** | |
| --- | --- | --- | --- | --- |
|  |  | **Scientific name** | **Genus** | **Species** |
| 1 | 930943 | *Sulfolobus islandicus* HVE10/4 | Sulfolobus | Sulfolobus islandicus |
| 2 | 1053692 | *Methanococcus maripaludis* X1 | Methanococcus | Methanococcus maripaludis |
| 3 | 573236 | *Bifidobacterium animalis* subsp. *lactis* V9 | Bifidobacterium | Bifidobacterium animalis |
| 4 | 862962 | *Bacteroides fragilis* 638R | Bacteroides | Bacteroides fragilis |
| 5 | 1218356 | *Chlamydia psittaci* WS/RT/E30 | Chlamydia | Chlamydia psittaci |
| 6 | 300852 | *Thermus thermophilus* HB8 | Thermus | Thermus thermophilus |
| 7 | 347495 | *Bacillus cereus* F837/76 | Bacillus | Bacillus cereus |
| 8 | 552536 | *Listeria monocytogenes* HCC23 | Listeria | Listeria monocytogenes |
| 9 | 1036673 | *Paenibacillus mucilaginosus* KNP414 | Paenibacillus | Paenibacillus mucilaginosus |
| 10 | 418127 | *Staphylococcus aureus* subsp. *aureus* Mu3 | Staphylococcus | Staphylococcus aureus |
| 11 | 226185 | *Enterococcus faecalis* V583 | Enterococcus | Enterococcus faecalis |
| 12 | 998820 | *Lactobacillus casei* BD-II | Lactobacillus | Lactobacillus casei |
| 13 | 299768 | *Streptococcus thermophilus* CNRZ1066 | Streptococcus | Streptococcus thermophilus |
| 14 | 536232 | *Clostridium botulinum* A2 str. Kyoto | Clostridium | Clostridium botulinum |
| 15 | 440085 | *Methylobacterium extorquens* CM4 | Methylobacterium | Methylobacterium extorquens |
| 16 | 395492 | *Rhizobium leguminosarum* bv. *trifolii* WSM2304 | Rhizobium | Rhizobium leguminosarum |
| 17 | 1117943 | *Sinorhizobium fredii* HH103 | Sinorhizobium | Sinorhizobium fredii |
| 18 | 1184253 | *Anaplasma phagocytophilum* str. HZ2 | Anaplasma | Anaplasma phagocytophilum |
| 19 | 449216 | *Rickettsia prowazekii* str. Rp22 | Rickettsia | Rickettsia prowazekii |
| 20 | 568706 | *Bordetella pertussis* 18323 | Bordetella | Bordetella pertussis |
| 21 | 122586 | *Neisseria meningitidis* MC58 | Neisseria | Neisseria meningitidis |
| 22 | 573059 | *Desulfovibrio vulgaris* RCH1 | Desulfovibrio | Desulfovibrio vulgaris |
| 23 | 367737 | *Arcobacter butzleri* RM4018 | Arcobacter | Arcobacter butzleri |
| 24 | 354242 | *Campylobacter jejuni* subsp. *jejuni* 81-176 | Campylobacter | Campylobacter jejuni |
| 25 | 402882 | *Shewanella baltica* OS185 | Shewanella | Shewanella baltica |
| 26 | 290339 | *Cronobacter sakazakii* ATCC BAA-894 | Cronobacter | Cronobacter sakazakii |
| 27 | 1104326 | *Enterobacter cloacae* subsp. *dissolvens* SDM | Enterobacter | Enterobacter cloacae |
| 28 | 373384 | *Shigella flexneri* 5 str. 8401 | Shigella | Shigella flexneri |
| 29 | 1123863 | *Pantoea ananatis* LMG 5342 | Pantoea | Pantoea ananatis |
| 30 | 768492 | *Serratia plymuthica* AS9 | Serratia | Serratia plymuthica |
| 31 | 434271 | *Actinobacillus pleuropneumoniae* serovar 3 str. JL03 | Actinobacillus | Actinobacillus pleuropneumoniae |
| 32 | 262728 | *Haemophilus influenzae* R2866 | Haemophilus | Haemophilus influenzae |
| 33 | 889738 | *Acinetobacter baumannii* MDR-TJ | Acinetobacter | Acinetobacter baumannii |
| 34 | 1042876 | *Pseudomonas putida* S16 | Pseudomonas | Pseudomonas putida |
| 35 | 441952 | *Francisella tularensis* subsp. *mediasiatica* FSC147 | Francisella | Francisella tularensis |
| 36 | 990315 | *Xanthomonas campestris* pv. *raphani* 756C | Xanthomonas | Xanthomonas campestris |
| 37 | 1133568 | *Brachyspira pilosicoli* B2904 | Brachyspira | Brachyspira pilosicoli |
| 38 | 1328311 | *Borrelia burgdorferi* CA382 | Borreliella | Borreliella burgdorferi |
| 39 | 243276 | *Treponema pallidum* subsp. *pallidum* str. Nichols | Treponema | Treponema pallidum |
| 40 | 1159202 | *Mycoplasma gallisepticum* NC06_2006.080-5-2P | Mycoplasma | Mycoplasma gallisepticum |

**Table S5: Reference genomes used for the second, the third and the fourth dataset. This list is referred from the experiment in Kraken 2.**

| **No** | **Taxonomy ID (species)** | **Species** | **NCBI Phylogeny Database** |
| --- | --- | --- | --- |
| 1 | 1280 | *Staphylococcus aureus* | Bacteria; Firmicutes; Bacilli; Bacillales; Staphylococcaceae; Staphylococcus |
| 2 | 1351 | *Enterococcus faecalis* | Bacteria; Firmicutes; Bacilli; Lactobacillales; Enterococcaceae; Enterococcus |
| 3 | 1423 | *Bacillus subtilis* | Bacteria; Firmicutes; Bacilli; Bacillales; Bacillaceae; Bacillus; Bacillus subtilis group |
| 4 | 1613 | *Lactobacillus fermentum* | Bacteria; Firmicutes; Bacilli; Lactobacillales; Lactobacillaceae; Lactobacillus |
| 5 | 1639 | *Listeria monocytogenes* | Bacteria; Firmicutes; Bacilli; Bacillales; Listeriaceae; Listeria |
| 6 | 287 | *Pseudomonas aeruginosa* | Bacteria; Proteobacteria; Gammaproteobacteria; Pseudomonadales; Pseudomonadaceae; Pseudomonas; Pseudomonas aeruginosa group |
| 7 | 28901 | *Salmonella enterica* | Bacteria; Proteobacteria; Gammaproteobacteria; Enterobacteriales; Enterobacteriaceae; Salmonella |
| 8 | 4932 | *Saccharomyces cerevisiae* | Eukaryota; Opisthokonta; Fungi; Dikarya; Ascomycota; saccharomyceta; Saccharomycotina; Saccharomycetes; Saccharomycetales; Saccharomycetaceae; Saccharomyces |
| 9 | 5207 | *Cryptococcus neoformans* | Eukaryota; Opisthokonta; Fungi; Dikarya; Basidiomycota; Agaricomycotina; Tremellomycetes; Tremellales; Tremellaceae; Filobasidiella; Filobasidiella/Cryptococcus neoformans species complex |
| 10 | 562 | *Escherichia coli* | Bacteria; Proteobacteria; Gammaproteobacteria; Enterobacteriales; Enterobacteriaceae; Escherichia |

**Table S6: The list of 10 species were presented Zymo mock community R10, which is generated on the Oxford Nanopore GridION.**

| **Species ID** | Minimap2 | Minimap2* | Kraken2 | Centrifuge | CLARK24 | CLARK31 | CDKAM |
| --- | --- | --- | --- | --- | --- | --- | --- |
| **1280** | 397609 | 410040 | 407545 | 405273 | 416180 | 407081 | 405819 |
| **1351** | 399959 | 422711 | 418962 | 425486 | 431269 | 421032 | 427635 |
| **1423** | 661800 | 673006 | 325040 | 493732 | 341239 | 300904 | 612361 |
| **1613** | 270512 | 274064 | 272157 | 275137 | 279293 | 272150 | 273070 |
| **1639** | 479115 | 506167 | 499895 | 511923 | 513955 | 501580 | 506396 |
| **287** | 41140 | 42379 | 39264 | 37253 | 39054 | 38149 | 40745 |
| **28901** | 52921 | 134862 | 131313 | 131245 | 132299 | 129189 | 135428 |
| **4932** | 87705 | 91567 | 86209 | 0 | 36169 | 34628 | 85025 |
| **5207** | 72526 | 76211 | 71403 | 0 | 70658 | 64681 | 71849 |
| **562** | 62202 | 144249 | 127870 | 129366 | 124456 | 117303 | 143648 |
| **Others** | 326323 | 76553 | 525190 | 490169 | 652871 | 243326 | 204129 |
| **Unclassified** | 397714 | 397714 | 344678 | 349942 | 212083 | 719503 | 343421 |

**Table S7: The number of reads classified by different classifiers on the Zymo mock community R10.**

**Others** category in Kraken2, Centrifuge, CLARK24, CLARK31, and CDKAM presents the number of incorrect classifications or classifications at taxonomy rank above the Species level. Meanwhile, **Others** category in Minimap2 indicates the number of read that could be mapped to multiple genomes among 10 species. Because there are several pairs of genomes sharing a part of DNA sequence such as Salmonella enterica (28901) and Escherichia coli (562) with same Family level, or Enterococcus faecalis (1351) and Lactobacillus fermentum (1613) with the same Order level, we create a new statistics Minimap2* for a higher resolution of Zymo community. In this statistics, we increase the counter by 0.5 per species for each read that can be mapped to 2 species by Minimap2.


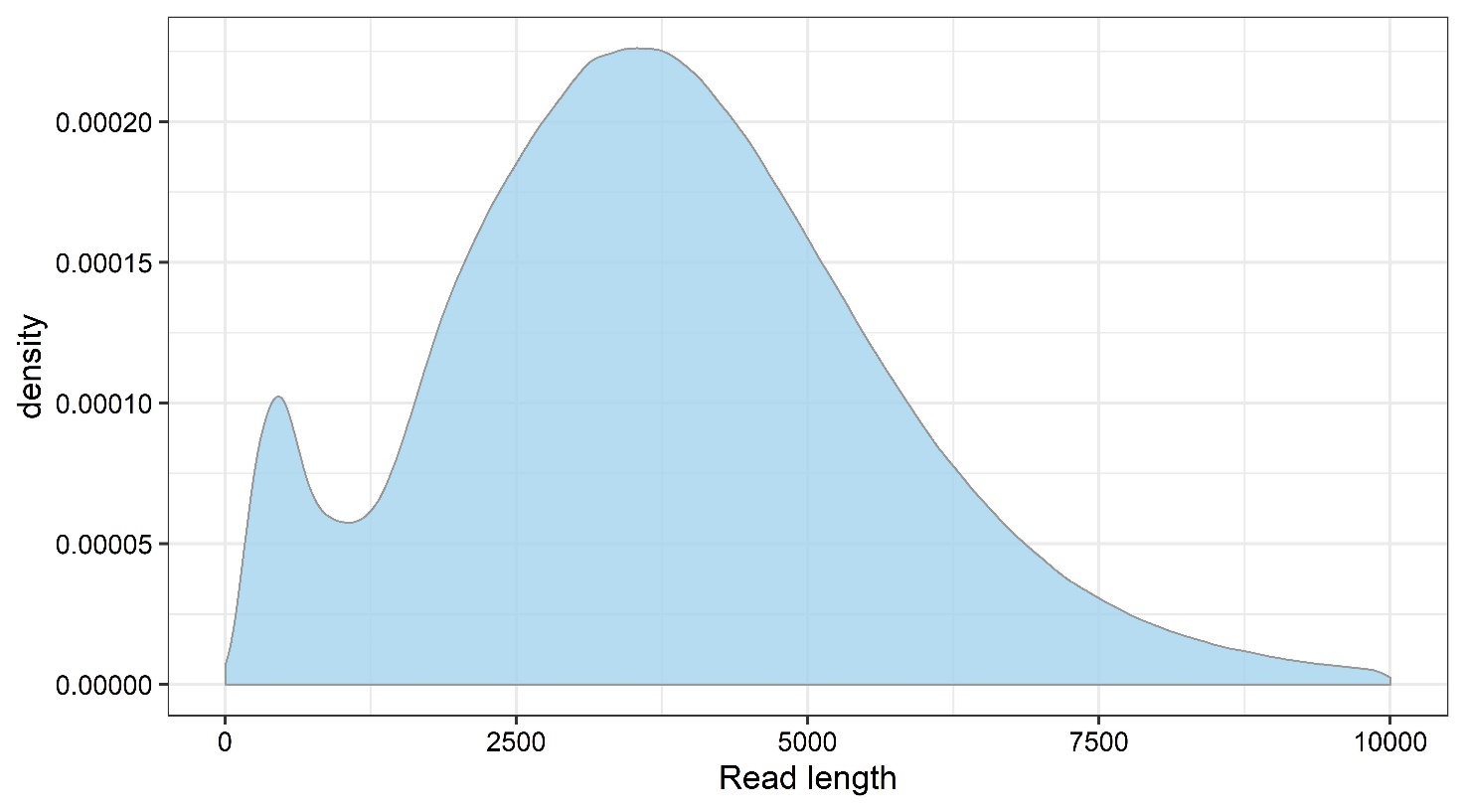


**Figure S1: The distribution of read length in Zymo R10 dataset.**


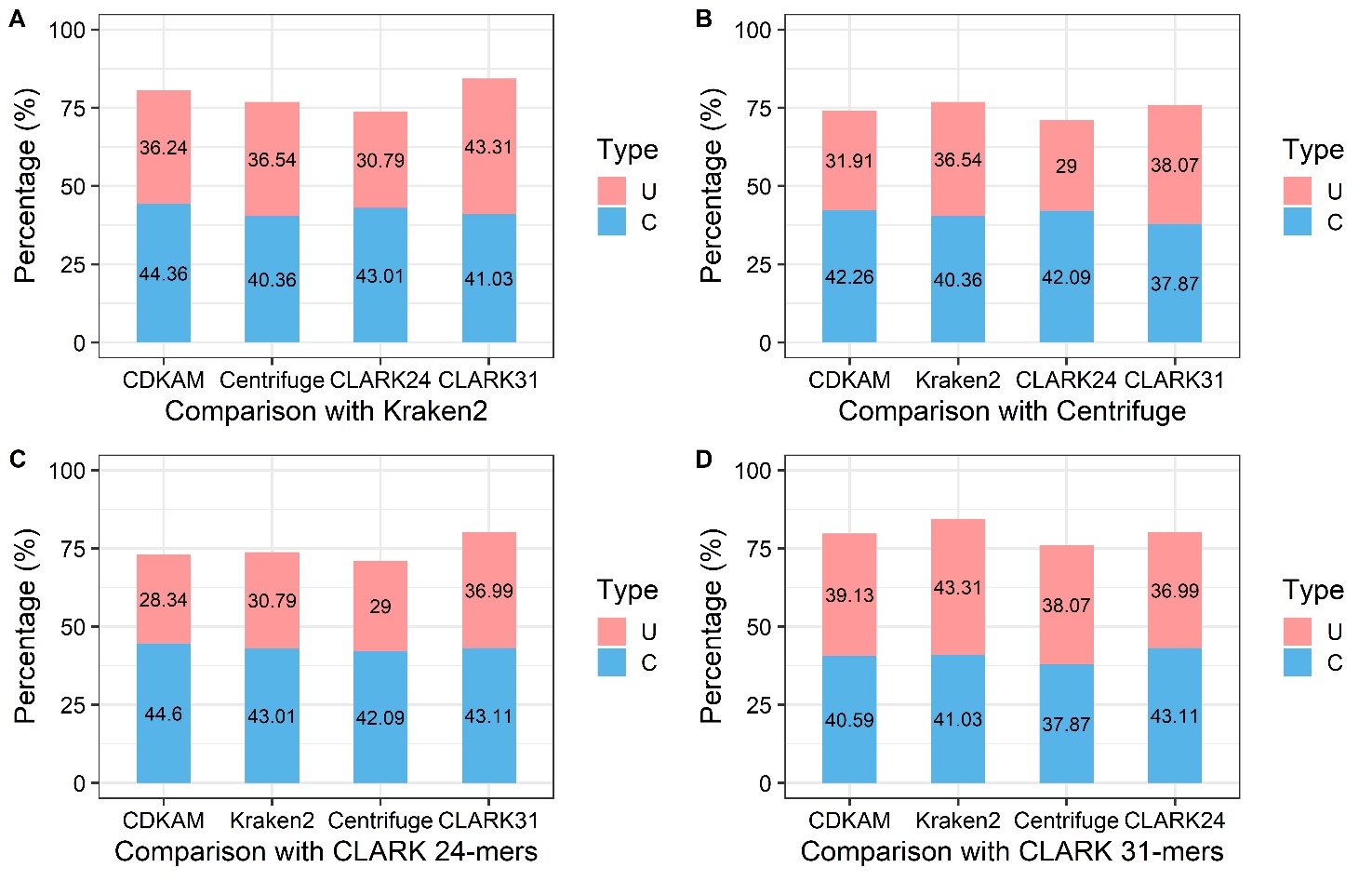


**Figure S2: Comparison of classifiers on the PRJNA493153 dataset by measuring the percentage of reads that have the same classification results.** Percentages shown in the figure are the number of unclassified (U) and classified (C) reads that are the consistent part of two classifiers.
